# Mental Summation of Temporal Duration within and across Senses

**DOI:** 10.1371/journal.pone.0141466

**Published:** 2015-10-27

**Authors:** Kohske Takahashi, Katsumi Watanabe

**Affiliations:** Research Center for Advanced Science and Technology, The University of Tokyo, Meguro, Tokyo, Japan; Duke University, UNITED STATES

## Abstract

Perceiving, memorizing, and estimating temporal durations are key cognitive functions in everyday life. In this study, a duration summation paradigm was used to examine whether summation of temporal durations introduces an underestimation or overestimation bias, and whether this bias is common to visual and auditory modalities. Two within- or across-modality stimuli were presented sequentially for variable durations. Participants were asked to reproduce the sum of the two durations (0.6–1.1 s). We found that the sum of two durations was overestimated regardless of stimulus modalities. A subsequent control experiment indicated that the overestimation bias arose from the summation process, not perceptual or memory processes. Furthermore, we observed strong positive correlations between the overestimation bias for different sensory modalities within participants. These results suggest that the sum of two durations is overestimated, and that supra-modal processes may be responsible for this overestimation bias.

## Introduction

How much time have you spent actually working today? To answer this question, you need to add the time spent at work between any breaks. This requires memorization and mental manipulation of time representations. Several models of time perception have been proposed [1–4]. Recent studies have also investigated how time is stored in memory [5–9]. Beyond time perception and memory, it is important to understand how time is mentally manipulated, because this will provide constraints on how time is represented in the brain. While knowledge of time perception and memory has increased, few studies have addressed mental manipulation of represented time (e.g., how two time durations are added) using timing tasks [10–14]. Therefore, the present study concentrated on summation of two temporal durations.

Summation is a basic mental manipulation regardless of stimulus dimension. The mental processes involved in summing two numbers have been explored in domains other than time perception. McCrink, Dehaene, & Dehaene-Lambertz [15] found that adding two numbers introduced an overestimation bias (*operational momentum* [16–18]). The mental summation of spatial representations (e.g., adding two line-lengths) has not been directly examined; however, line bisection modulates line-length perception, which suggests that perceiving the whole from two parts is biased. For example, Charras & Lupiáñez [19,20]reported that symmetrically bisected lines were underestimated, while asymmetrically bisected lines were overestimated. Given these biases in summing numerical and spatial magnitudes, it is intriguing to ask whether common processes underlie mental summation in all modalities. Recent studies have suggested that humans may use common cortical mechanisms to quantitatively represent time, space, and number (A Theory of Magnitude; AToM [21]). Investigating the processes involved in mental summation of magnitudes would allow us to extend the concept of AToM from representation to mental manipulation. To this end, here we investigated mental summation of temporal duration.

The present study also addressed whether stimulus modality affects mental summation of time. Previous work has examined whether time perception is mediated by sensory-specific processes or by a supra-modal process, with some studies demonstrating sensory-specific characteristics of time perception [3,22–27]. For example, adaptation to a visual duration leads to an aftereffect for perception of subsequent visual, but not auditory, durations [23]. Modality-specific processes have also been implicated for time memory. Secondary visual working memory tasks decrease concurrent visual, but not auditory, memory performance[27]. In addition, decay of time memory depends on sensory modality [9]. These modality-specific effects support the existence of distributed clocks in the brain [4]. Therefore, it is possible that modality-specific processes are involved in mentally summing time.

The present study had two aims: (1) To investigate whether summing temporal durations would introduce overestimation bias; (2) To examine whether this bias is common to different sensory modalities. To this end, we asked participants to reproduce the sum of two durations, each of which was presented in either the visual or auditory modality. If under- or overestimation biases are comparable between modalities, it would imply that a common process underlies mental summation of temporal duration regardless of stimulus modality. We also examined individual differences in estimation biases, specifically whether bias magnitudes were correlated between modality conditions, to further examine modality dependencies in mental summation.

## Experiment 1

### Methods

#### Participants

Twenty-two volunteers (15 male and 7 female, mean age = 21.0 years, SD = 1.62) participated after providing written informed consent. All participants had normal or corrected-to-normal visual and auditory abilities. The study was approved by the Ethics Committee of the University of Tokyo and run in accordance with the Declaration of Helsinki.

#### Apparatus and stimuli

Participants sat in a dark and quiet room. Visual stimuli were presented on a cathode ray tube monitor (refresh rate = 100 Hz) at a viewing distance of 57 cm. Auditory stimuli were presented through headphones (HDA 200, Sennheiser). The experiments were run on an Apple Mac mini with MATLAB and the Psychophysics Toolbox extension [28–30]. The visual stimulus was a white circle (2.1° radius) presented in the center of a black screen. The auditory stimulus was a 756 Hz pure tone including a 10 ms ramp at onset and offset.

#### Procedure

The experiment consisted of a single-stimulus session and a subsequent summation session. In the single-stimulus session, trials started with a blank display for 0.5–1.0 s, after which either a visual or auditory stimulus was presented. After the stimulus disappeared, participants reproduced the stimulus duration by pressing and releasing the space bar. No feedback was given. Stimulus duration was 0.6, 0.7, 0.8, 0.9, 1.0, or 1.1 s. Each stimulus duration was presented 5 times. There were 30 auditory and 30 visual trials that were presented in a pseudorandom order.

In the summation session, a blank display was followed by the first stimulus. After a random inter-stimulus interval (ISI) of 0.5–0.7 s, the second stimulus appeared. Then, participants were asked to reproduce the sum of the first and second stimulus durations by pressing and releasing the space bar. No feedback was given. The sum of the two durations was 0.6, 0.7, 0.8, 0.9, 1.0, or 1.1 s. The relative durations of the first and second stimuli were either 0.4 versus 0.6 (e.g., when the sum of the stimuli was 0.6 s, the durations of the first and second stimuli were 0.24 s and 0.36 s, respectively), 0.5 versus 0.5, or 0.6 versus 0.4. The first and second stimuli were either visual–visual (VV), auditory–auditory (AA), visual–auditory (VA), or auditory–visual (AV). There were four trials for each combination of modality, relative duration, and total duration for a total of 288 trials (4 repetitions × 4 modality combinations × 3 relative durations × 6 total durations). The trial sequence was pseudorandom.

#### Data Analysis

First, we calculated normalized duration estimates for each trial by dividing the reproduced duration by the stimulus duration (for the single-stimulus session) or by the actual sum of stimulus durations (for the summation session). Outlier detection was performed for each participant for each task. An outlier was defined as a normalized duration estimate that was more than three standard deviations from the participants’ mean, and these trials were excluded from the analyses. We then calculated mean normalized duration estimates for each condition for each participant. Results of the single-stimulus session were used to test the hypothesis that summation would introduce no bias. Summation bias was defined as the difference between predicted and observed means.

### Results and discussion

**Fig 1:**
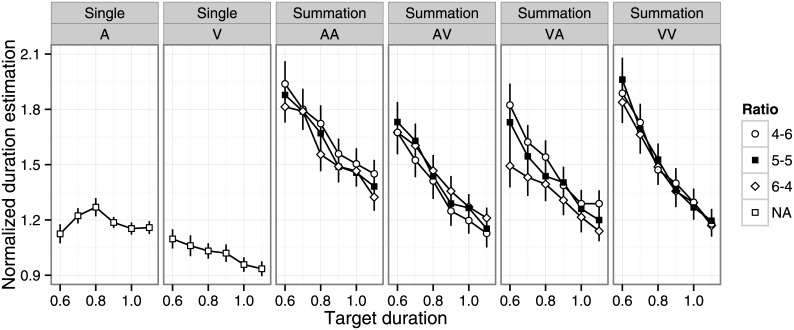
Mean normalized duration estimates as a function of target duration, duration ratio, and modality condition. Error bars indicate standard error of the mean. A = auditory; V = visual; AV = auditory–visual; VA = visual–auditory; AA = auditory–auditory; VV = visual–visual.

Outliers were rare in the single-stimulus (0.5%) and summation (0.8%) tasks. First, we examined the effects of target duration, duration ratio, and modality condition (Fig 1, see also S1 Table). For the single-stimulus task, a two-way repeated measures ANOVA revealed significant main effects of target duration (F(5, 105) = 6.84, p < .001, η_p_^2^ = .26) and modality (F(1, 21) = 40.7, p < .001, η_p_^2^ = .66), and a significant interaction (F(5, 105) = 4.27, p < .01, η_p_^2^ = .17). The auditory stimulus was estimated as longer than the visual stimulus, consistent with previous findings [31]. The effect of duration depended on modality condition: normalized duration estimates were shorter for longer target durations for visual stimuli (significant pairwise difference was observed for 0.6 > 1.0, 1.1; 0.7 > 1.0, 1.1; 0.8 > 1.1, multiple comparisons using Ryan’s methods with α = .05; the same correction method is used for multiple comparisons in the remainder of the paper), while normalized duration estimates were longer for the middle range of target durations for auditory stimuli (0.6, 1.0, 1.1 < 0.8, α = .05).

**Fig 2:**
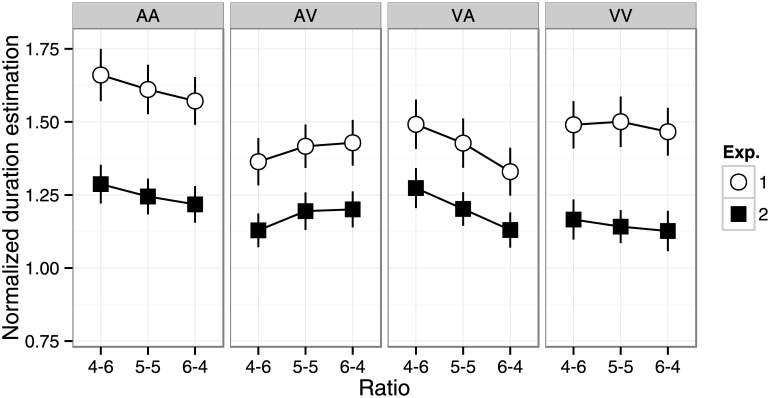
Normalized duration estimates in the summation task (Experiment 1) and dual-stimulus task (Experiment 2) as a function of duration ratio. Error bars indicate standard error of the mean.

For the summation task, a three-way repeated measures ANOVA revealed significant main effects of target duration (F(5, 105) = 104.7, p < .001, η_p_^2^ = .83), duration ratio (F(2, 42) = 8.38, p < .001, η_p_^2^ = .29), and modality condition (F(3, 63) = 24.8, p < .001, η_p_^2^ = .54), and significant target duration × modality (F(15, 315) = 4.66, p < .001, η_p_^2^ = .19) and duration ratio × modality (F(6, 126) = 9.94, p < .001, η_p_^2^ = .32) interactions. Normalized duration estimates were longer for shorter target durations in all conditions, but the magnitude of the effect depended on modality condition. Normalized duration estimates were shortest in the AV and VA conditions, followed by the VV condition, and then the AA condition. The effects of duration ratio were qualitatively different across modality conditions (Fig 2, AA: 4–6 > 5–5, 6–4; AV: 4–6 < 6–4; VA: 4–6 > 5–5 > 6–4; VV: n.s. α = .05).

**Fig 3:**
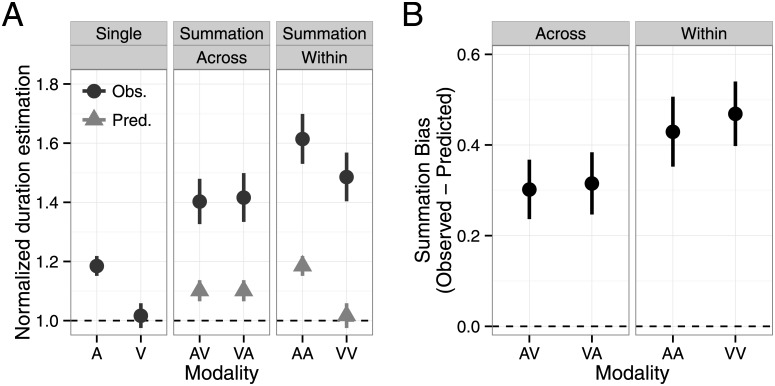
The summation bias observed in Experiment 1. (A) Normalized duration estimates as a function of modality condition. The observed data (black) and predictions based on single-stimulus task performance (gray) are shown. (B) The summation bias is defined as the difference between observed and predicted data. Error bars indicate standard error of the mean. A = auditory; V = visual; AV = auditory–visual; VA = visual–auditory; AA = auditory–auditory; VV = visual–visual; obs. = observed; pred = predicted.

Fig 3 shows mean normalized duration estimates and summation biases as a function of modality condition. In the summation session, we observed a significant overestimation biases in all modality conditions (Fig 1B, one-sample t-test, AA: t(21) = 5.58, p < .001, d’ = 2.43; AV: t(21) = 4.62, p < .001, d’ = 2.02; VA: t(21) = 4.60, p < .001, d’ = 2.01; VV: t(21) = 6.60, p < .001, d’ > 2.88). A one-way repeated measures ANOVA revealed a statistically significant effect of modality condition (F(3, 63) = 18.4, p < .001, η_p_^2^ = .47). We also found that the overestimation bias was larger for within- than across-modality summation (a one-way ANOVA with averaged AA and VV data as within-modality and averaged AV and VA data for across-modality, F(1, 21) = 37.6, p < .001, η_p_^2^ = .64).

Participants might perform the duration summation task by measuring the elapsed time from the onset of the first stimulus to the offset of the second stimulus instead of mentally summing the two durations. This strategy would lead to an overestimation bias due to the ISI. If this is the case, ISI should be correlated with the difference between the reproduced and actual stimulus duration; however, this correlation was not significant for any participants (correlation between ISI and the difference between the reproduced and actual duration instead of the normalized duration, mean r = .03).

**Fig 4:**
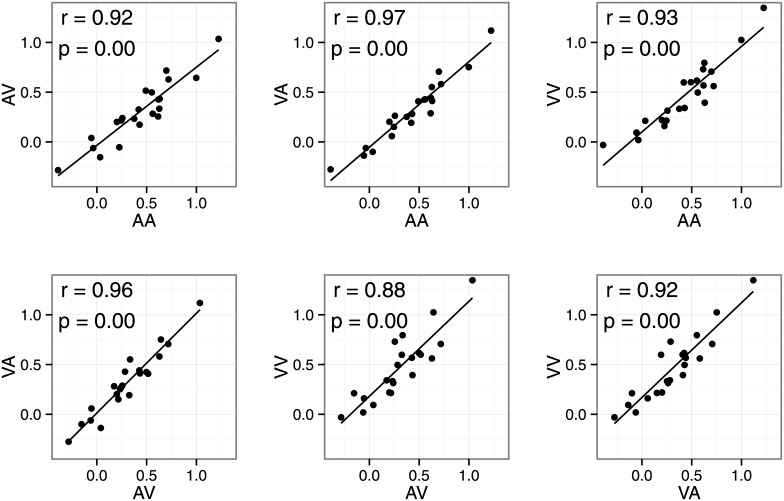
Scatter plots of the summation bias in Experiment 1. Each point represents data from one participant.

Fig 4 shows within-subject correlations between the summation bias for different modality conditions in the summation session. We found strong correlations (r > .87) between all modality conditions. Furthermore, we tested the equality of two correlation coefficients for all 15 pairs (e.g., r for AA/AV vs. r for VV/VA) using the cocor R package (tested by Hittner, 2003 and Silver, 2004, α = .05). There were no significant differences in correlation coefficients between any pairs. This suggests that a common process generated the overestimation bias regardless of modality condition. Note that correlations between overestimation biases cannot be attributed to individual differences in manual response generation in the duration reproduction task. The summation bias was defined as the difference between observation and prediction. Because the prediction was based on data from the single-stimulus session, the prediction also includes response-related processes. Because the summation bias was computed by subtracting the predicted value from the observed value, it cannot reflect response-related biases. This is supported by the data, as we did not observe any within-subject correlations (mean r = .07) between normalized duration estimates in the single-stimulus session and the summation bias in the summation session. These results suggest that the overestimation bias is a supra-modal mental summation process. However, it is possible that common subtrahends (i.e., normalized duration estimates in the single-stimulus session) inflated correlation coefficients in some pairs. For example, in the VA-AV correlation, we subtracted normalized duration estimates for the A and V conditions from the VA and AV conditions. This is known to inflate correlation coefficients (Miller & Ulrich, 2013). Therefore, the correlation coefficients must be interpreted with caution, as the true correlation between overestimation biases might be smaller than those observed.

## Experiment 2

Experiment 1 demonstrated that reproducing the sum of two temporal durations led to an overestimation bias compared with reproducing single temporal durations. However, the single-stimulus and summation tasks in Experiment 1 differed in some important ways. For example, the summation task required perceiving, encoding, and retaining two stimulus durations, while only one duration had to be processed in the single-stimulus task. Therefore, we cannot rule out the possibility that differences in these processes might be responsible for the overestimation bias, rather than mental summation. To examine this possibility, in Experiment 2 the stimuli were identical to Experiment 1, such that participants needed to perceive, encode, and retain two durations, but mental summation was not required. That is, participants reproduced the two durations one at a time instead of reproducing their sum. If an overestimation bias is also observed in this experiment, it would suggest that the overestimation is the outcome of processing two durations. In contrast, if the summation bias is absent, this would suggest that the bias observed in Experiment 1 was due to mental summation.

### Methods

Twenty-two new volunteers (17 male and 5 female, mean age = 20.8, SD = 1.74) were recruited. The apparatus, stimuli, and procedure were identical to Experiment 1, except for the task in the dual-stimulus session. In the dual-stimulus session, the stimuli were identical to those in the summation session of Experiment 1, and participants reproduced the duration of the first stimulus and then reproduced the duration of the second stimuli. Normalized duration estimates were computed by dividing the sum of the two reproduced durations by the sum of the two stimulus durations.

### Results and discussion

**Fig 5:**
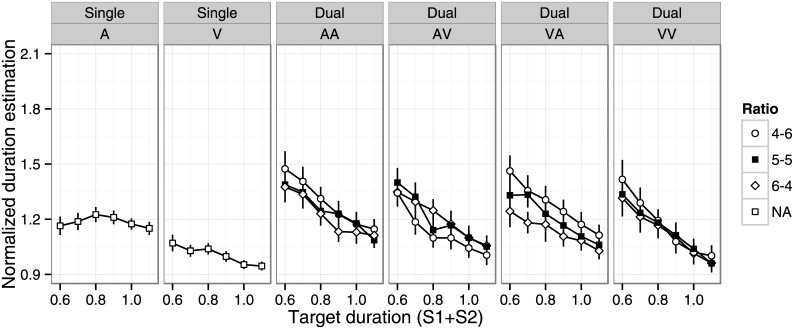
Mean normalized duration estimates as a function of target duration, duration ratio, and modality condition. Error bars indicate standard error of the mean.

Outliers were rare in the single-stimulus (0.6%) and dual-stimulus (1.9%) tasks. The results for the single-stimulus condition were similar to Experiment 1 (Fig 5, see also S1 Table). We observed significant main effects of target duration (F(5, 105) = 3.76, p < .05, η_p_^2^ = .20) and modality (F(1, 21) = 57.1, p < .001, η_p_^2^ = .73), and a significant interaction (F(5, 105) = 3.32, p < .01, η_p_^2^ = .13). The auditory stimulus was estimated as longer than the visual stimulus. Normalized duration estimates were shorter for the longer target duration for visual stimuli (significant pairwise difference for 0.6 > 0.9 1.0, 1.1; 0.7 > 1.0, 1.1; 0.8 > 1.1, α = .05), whereas there was no effect of target duration for auditory stimuli.

For the dual-stimulus task, we performed a three-way repeated measures ANOVA with target duration (i.e., sum of two stimulus durations), duration ratio, and modality condition as factors. We observed significant main effects of target duration (F(5, 80) = 56.1, p < .001, η_p_^2^ = .73), duration ratio (F(2, 42) = 10.6, p < .001, η_p_^2^ = 0.34), and modality condition (F(3, 63) = 11.0, p < .001, η_p_^2^ = .34). There were also significant target duration × duration ratio (F(10, 210) = 2.94, p < .01, η_p_^2^ = .12) and duration ratio × modality condition (F(6, 126) = 16.6, p < .001, η_p_^2^ = .44) interactions. Normalized duration estimates were longer for shorter target durations for all conditions. Normalized duration estimates were longer in the AA than AV and VV conditions. The effect of duration ratio was qualitatively different between modality conditions (Fig 2, AA: 4-6 > 5-5, 6-4; AV: 4-6 < 5-5, 6-4; VA: 4-6 > 5-5 > 6-4; VV: n.s., α = .05). It is noteworthy that the effect of duration ratio was similar between Experiment 1 and 2 (Fig 2). We performed a mixed four-way ANOVA with task (summation vs. dual-stimulus), duration ratio, target duration, and modality condition as factors. The main effect of task was significant (F(1, 42) = 8.08, p < .01, η_p_^2^ = .16), such that normalized duration estimates were larger in Experiment 1 than Experiment 2. The effects of duration ratio (F(2, 84) = 17.9, p < .001, η_p_^2^ = .30), modality (F(3, 126) = 29.9, p < .001, η_p_^2^ = .42), and target duration (F(5, 210) = 160.5, p < .001, η_p_^2^ = .79) were also significant. The task × duration ratio (F(2, 84) = 0.34, p = .71, η_p_^2^ = .01), task × duration ratio × modality (F(6, 252) = 0.40, p = .87, η_p_^2^ = .01), task × duration ratio × target duration (F(10, 420) = 1.08, p = .38, η_p_^2^ = .03), duration ratio × modality × target duration (F(30, 1260) = 1.11, p = .34, η_p_^2^ = .03), and four-way interactions (F(30, 1260) = 0.89, p = .59, η_p_^2^ = .02) were not significant. These results suggest that duration ratio influenced reproduced duration independent of modality and task. Furthermore, the significant task × modality interaction (F(3, 111) = 7.96, p < .001, η_p_^2^ = .18) implies that the difference between tasks was larger for AV and VA versus AA and VV conditions, consistent with the analysis of summation bias in Experiment 1 (Fig 3). The effects of duration ratio depended on stimulus interval in Experiment 2. We addressed this further by analyzing the first and second intervals separately. All two-way interactions with target duration were significant (target duration × task: F(5, 210) = 11.8, p < .001, η_p_^2^ = .22; target duration × duration ratio: F(10, 420) = 3.13, p < .01, η_p_^2^ = .07; target duration × modality: F(15, 630) = 5.79, p < .001, η_p_^2^ = .12). The target duration × task × modality was also significant (F(15, 630) = 1.83, p < .05, η_p_^2^ = .04). The other three- and four-way interactions were not significant.

**Fig 6:**
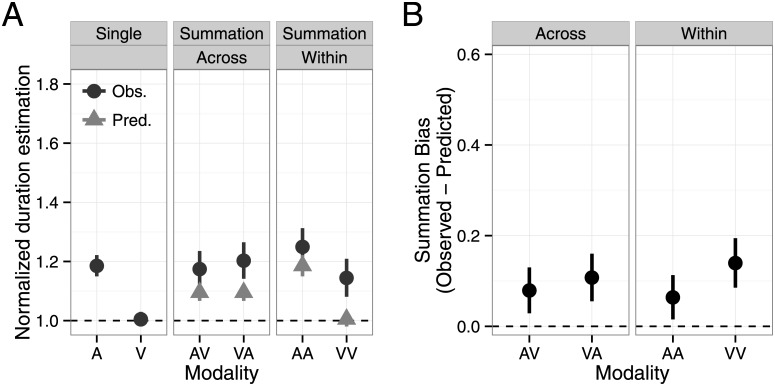
Biases observed in Experiment 2. (A) Normalized duration estimates as a function of modality condition. The observed data (black) and predictions based on single-stimulus task performance (gray) are shown. (B) The bias is defined as the difference between observed and predicted data. Error bars indicate standard error of the mean. A = auditory; V = visual; AV = auditory–visual; VA = visual–auditory; AA = auditory–auditory; VV = visual–visual; obs. = observed; pred = predicted.

Fig 6A shows mean normalized duration estimates and Fig 6B shows summation biases as a function of modality condition. There was a slight tendency toward overestimation in the dual-stimulus task (Fig 6B; AV: t(16) = 1.49, p = .17, d’ = 0.74, VA: t(16) = 1.96, p = .067, d’ = 0.86, AA: t(16) = 1.65, p = .12, d’ = 0.83, VV: t(16) = 2.30, p = .035, d’ = 1.15). However, the magnitude of the overestimation bias was much smaller in the dual-stimulus compared with summation task. The comparison between experiments (mixed two-way modality condition × task ANOVA, Fig 3B vs. Fig 6B) revealed a significant main effect of task (F(1, 37) = 7.66, p < .01, η_p_^2^ = .17). The effect of modality condition on the dual-stimulus bias was statistically significant (F(3, 48) = 3.06, p < .05, η_p_^2^ = .16). However, unlike the overestimation bias in the summation task, we did not find any significant differences in the pairwise comparisons. Furthermore, the magnitude of the overestimation biases was comparable in within- and across-modality conditions (F(1, 16) = 0.92, p = .35, η_p_^2^ = .05).

The slight overestimation bias in Experiment 2 might be due to differences in the range of reproduced durations between the single-stimulus session (0.6–1.1 s) and the dual-stimulus session (0.24–0.66 s). For example, Shi, Ganzenmüller, and Müller (2013) observed an overestimation bias in duration reproduction of a single auditory stimulus, in which shorter durations led to larger overestimation biases. In our study, we also found that shorter target durations led to longer normalized duration estimates (Figs 1 and 5). To further test this claim, we compared normalized duration estimates in a subset of the data. In Experiment 2, we presented targets for 0.6 s in both single and dual (duration ratio 0.6 for a total duration of 1.0 s) conditions. As expected, there was no overestimation bias (normalized duration estimates: auditory single = 1.17; visual single = 1.12; auditory dual = 1.18; visual dual = 0.90).

**Fig 7:**
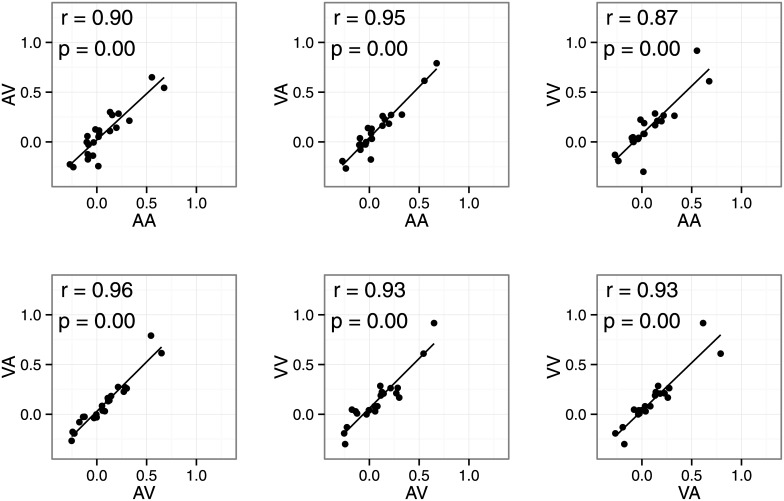
Scatter plots of the bias in Experiment 2. Each point represents data for one participant.

Fig 7 shows the within-subject correlations between dual-stimulus biases. We found strong correlations (r > .87) between all modality conditions, consistent with Experiment 1. Correlation coefficients did not differ between any pairs. Thus, the dual-stimulus bias also seems to originate from supra-modal processes.

#### Effect of interval

**Fig 8:**
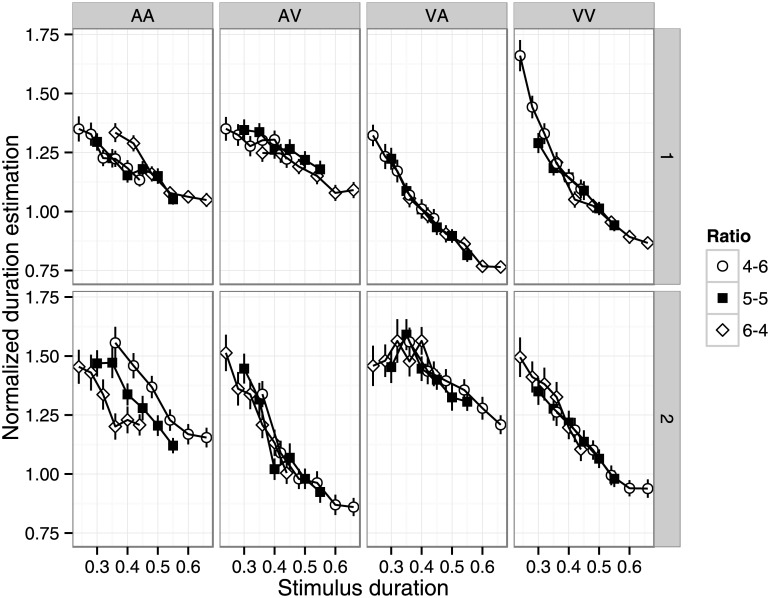
Normalized duration estimates in the dual-stimulus task as a function of stimulus duration, duration ratio, interval, and modality condition. Error bars indicate standard error of the mean.

**Fig 9:**
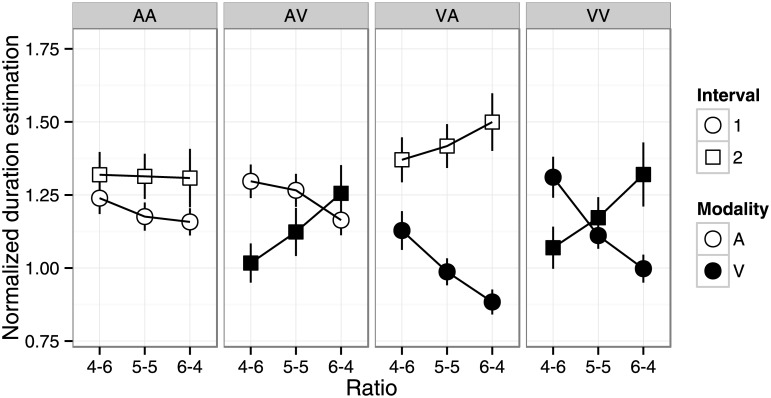
Normalized duration estimates in the dual-stimulus task as a function of duration ratio, interval, and modality condition. Error bars indicate standard error of the mean.

In the dual-stimulus task, participants reproduced two stimulus durations on each trial. We examined the effects of interval on duration reproduction. We computed the normalized duration estimates for the first and second intervals separately by dividing the first and second reproduced durations by the first and second stimulus durations, respectively. Fig 8 shows the effects of stimulus duration for the first and second intervals. Visual inspection suggests that shorter target durations resulted in longer duration estimates. Fig 9 shows the effects of interval, modality condition, and duration ratio. A four-way repeated measures ANOVA (modality, interval, duration ratio, and target duration) revealed significant main effects of modality (F(3, 63) = 7.71, p < .001, η_p_^2^ = .27), interval (F(1, 21) = 9.86, p < .01, η_p_^2^ = .32), and target duration (F(5, 105) = 52.7, p < .001, η_p_^2^ = .71). All two-way, three-way, and four-way interactions were significant, expect for interval × target duration and modality × duration ratio × target duration (modality × ratio: F(6, 126) = 5.69, p < .001, η_p_^2^ = .21; modality × interval: F(3, 63) = 42.5, p < .001, η_p_^2^ = .67; ratio × interval: F(2, 42) = 26.0, p < .001, η_p_^2^ = .55; modality × target duration: F(15, 315) = 2.42, p < .05, η_p_^2^ = .10; ratio × target duration: F(10, 210) = 2.45, p < .05, η_p_^2^ = .11; modality × ratio × interval: F(6, 126) = 25.0, p < .001, η_p_^2^ = .54; modality × interval × target duration: F(15, 315) = 9.43, p < .001, η_p_^2^ = .31, interval × ratio × target duration: F(10, 210) = 3.23, p < .01, η_p_^2^ = .13, four-way: F(30, 630) = 1.98, p < .01, η_p_^2^ = .09). While the patterns may appear complicated, the effects can be largely explained by two factors. First, the auditory stimulus was perceived as longer than the visual stimulus, resulting in longer estimates for the first (second) interval than for the second (first) interval in the AV (VA) condition. Second, shorter stimulus durations were estimated as longer (Fig 8), resulting in a positive and negative slope for the first and second interval, respectively, in the VA, VA, and VV conditions. The results of the AA condition were rather puzzling. In particular, the second interval in the AA condition (left-bottom panel of Fig 8) clearly shows an interaction between target duration and duration ratio. This might reflect interference from the first interval when two auditory durations need to be encoded.

## General Discussion

The present study provides some empirical findings regarding mental summation of temporal durations. The primary observation is that mental summation introduced an overestimation bias. The overestimation bias was observed regardless of the sensory modalities being summed. Because the duration of the blank ISI did not correlate with reproduced durations, the overestimation bias cannot be attributed to an artifact where participants simply reproduced the duration from the onset of the first stimulus to the offset of the second stimulus rather than summing the two durations. The control experiment showed that the overestimation bias was reduced when mental summation was not required; perceiving, encoding, and retaining two durations was not sufficient to generate an overestimation bias comparable to that observed when two durations were summed.

Timing processes in the human brain have been of interest to researchers for a long time. In particular, recent studies have investigated timing processes in terms of a complicated interaction between various cognitive mechanisms, and have challenged various issues related to memory, modality effects, attention, and perceptual learning [32,33]. The present study provides a new constraint by addressing mental manipulation and modality effects on temporal duration. Integrating these studies could contribute to understanding timing processes in the human brain. For example, the modality specificity of timing processes is controversial [33]; some aspects of time perception show modality-specific encoding of timing information, while others show modality-independent use of time or duration. Our results suggest that mental summation of duration takes place in a supra-modal manner. An integrated view of these studies suggests that modality-specific encoding of time is translated into a supra-modal representation that can be submitted to supra-modal manipulation processes.

Before discussing the mental summation process in detail, we address some methodological issues that may limit the validity and scope of the theoretical discussion. First, in our experiments participants performed the single-stimulus condition first, followed by the summation condition (Experiment 1) or dual-stimulus condition (Experiment 2). Thus, condition order may have influenced the overestimation bias. The larger overestimation bias in Experiment 1 versus Experiment 2 indicates that the summation process introduced an extra overestimation bias. Furthermore, we tested the opposite order (summation condition followed by the single-stimulus condition) with a small group of participants (N = 5) and obtained a similar overestimation bias (mean bias: Experiment 1 = 0.38; Experiment 2 = 0.10; summation with opposite session order = .23). Thus, the bias cannot be explained by order effects alone. Nevertheless, further investigation of the interaction between condition order and overestimation bias would be helpful for quantitative evaluation of the overestimation bias in duration summation.

Second, our argument that the overestimation bias was larger in the summation than dual-stimulus condition relied on an across-experiment contrast. A within-experiment design would be preferable for highlighting the specific effects of summation.

Third, the stimuli were identical across experiments. Consequently, the reproduced duration range was shorter in the dual-stimulus versus single stimulus condition in Experiment 2. Although we briefly mentioned this issue above, it would be better to compare single-stimulus conditions with durations that match those in dual-stimulus conditions. These issues can be addressed in future experiments and will help provide a clearer and more complete picture of mental summation of temporal duration.

Fourth, we used the reproduced duration in the single-stimulus condition as a baseline, and the stimulus duration in the single-stimulus condition matched the sum of the two stimulus durations in the summation session. In other words, each stimulus duration in the summation condition was shorter than in the single-stimulus session. Based on the negatively accelerating growth of subjective time shown in Figs 1, 5 and 8, this manipulation may have inflated the overestimation bias because subjective duration for the sum of shorter stimuli should be longer than duration for one longer stimulus, even if actual durations are identical. Although this account cannot explain the larger overestimation bias in the summation versus dual-stimulus condition, part of the overestimation bias (i.e., sum–single) may arise from this negatively accelerating growth. Therefore, the magnitude of the overestimation bias needs to be interpreted with caution.

### Consistency and discrepancy with previous studies

Although the summation process has not been directly studied, Fortin and colleagues investigated time perception using similar stimulus conditions as the present study [11–14]. In their studies, participants were required to produce a target duration. A visual or auditory stimulus was presented with a break that varied in duration. Participants need to suspend timing during the break period and resume timing when the stimulus resumed. Thus, participants needed to sum the pre-break and post-break durations. Fortin and Massé [11] and Viau-Quesnel et al. [14]directly compared with- and without-break conditions, which were similar to the summation and single-stimulus tasks in the present study, respectively. They reported that, in most cases, the reproduced duration was shorter in the with- versus without-break condition. These results indicate overestimation of duration when there was a break, consistent with the present study.

They also reported that produced duration was shorter than the target duration in almost all cases. These results indicate a shortening of subjective duration compared to target duration, which is inconsistent with the present study. This inconsistency may be due to differences between the timing tasks. In the production task with simultaneous stimulus presentation used by Forting and colleagues, participants were required to process duration accumulation online because they needed to press a key as soon as possible when the elapsed time reached the target duration. In contrast, in the reproduction task used in our study, participants needed to encode two durations separately, retain them in memory, and then sum them off-line. This might lead to different summation strategies: online suspend-and-resume timing versus offline encoding-and-summing durations. Moreover, the production task might be influenced by action preparation (e.g.,[34]), because participants need to perceive the stimulus and generate a motor action. In the reproduction task, participants do not need to generate a motor action while perceiving the stimuli, and stimuli are not presented when they make their motor response. Taken together, comparing the single-stimulus (or without-break) versus summation (or with-break) durations demonstrates a robust overestimation bias, but there are some discrepancies between the target and (re)produced durations.

### Effects of duration ratio (break location effect)

Fortin and colleagues demonstrated robust break location effects [11–14]: a longer pre-break period resulted in a longer produced duration (underestimation) than a shorter pre-break period. They explained the break location effect in terms of attentional allocation to the upcoming event. In the pre-break period, participants need to count time while simultaneously attending to the stimulus break. Attention to the external upcoming event may detract attention from the timing task. Because attended objects are perceived as having longer durations [2,3,35,36], a longer pre-break duration results in duration underestimation. Break location was also manipulated in the present study (duration ratio of the first and second intervals), but there were no clear break location effects (Fig 2). The results in the AA condition showed a similar pattern, that is, a longer pre-break duration (6–4 ratio) resulted in slight underestimation (shorter reproduction). In the VV condition, the effect was not significant. However, this discrepancy may be consistent with and support the attentional account of the break location effect. In Fortin and colleagues’ time production task, attention allocation to the upcoming event was only required during the pre-break, but not post-break, period. In the present study, participants were required to attend to the upcoming external event, i.e., stimulus offset, in both intervals. Thus, attention is detracted from the timing task in both intervals, nullifying the break location effect.

### Relation to the weighted sum of segments model

Recently, Matthews [37] proposed the weighted sum of segments model to explain perception of temporal durations that consist of several segments. This computational model has successfully explained some behavioral results quantitatively [10,37]. In these studies, participants judged the duration of a stimulus that consisted of multiple segments. Although participants were not explicitly instructed to sum multiple durations, the model describes how multiple segments are summed into one duration. According to this model, when summing multiple segments, more recent segments are weighted more heavily, leading to a specific prediction for the effects of duration ratio: the reproduced duration should be shorter in trials where the first duration is longer than the second (i.e., 6–4 condition) compared to trials where the first duration is shorter than the second duration. However, the effects of duration ratio in the present study were not perfectly consistent with this prediction. Reproduced durations were longer in the 4–6 versus 6–4 AA conditions, while effects of duration ratio were not clear in the VV condition (Fig 2). This inconsistency may be due to differences in stimulus conditions, in particular the presence of a break between segments. Participants in the present study needed to sum two durations separated by a short break, while participants in previous studies [10,37] needed to (likely implicitly) sum segments of one stimulus separated by a transient change (e.g., change of tone). Weights for the first and second durations in the present study would not be asymmetrical if breaks reset the recency weighting effect. Thus, the present study may provide constraints that could extend the weighted sum of segments model to handle the sum of temporally separated events.

### Modality effects in duration summation

We also investigated whether stimulus modality influences mental summation of temporal duration. We found an overestimation bias regardless of sensory modalities. Furthermore, there were strong correlations between the overestimation bias for all modality pairs (r > 0.87, Fig 2). These results favor a supra-modal process rather than sensory-specific processes. There were also strong correlations between the dual-stimulus bias in Experiment 2 (Fig 4). Maintaining two (or more) durations may introduce additional individual differences that are unrelated to sensory modality. Although some of the individual differences in the summation bias might reflect those maintenance processes, our results suggest that there are additional supra-modal processes for mental summation of temporal durations.

If sensory-specific processes play a role in duration summation, the summation bias would be a combination of the supra-modal bias involved in maintaining two durations and a sensory-specific bias involved in summing two durations, leading to weaker correlations between summation versus dual-stimulus biases; this is not what we observed, and we did not find any signatures of sensory-specific processes for duration summation. Some signatures of sensory-specific processing have been found for duration perception and memory [3,9,22–27]. Time summation seems to be performed at a higher (i.e., later) stage, and presumably involves a more abstract form of time representation than perception and memory.

Unexpectedly, we observed a difference between within- and across-modality conditions in Experiment 1: The overestimation bias was larger for within- versus across-modality summation. This difference was likely introduced during summation because we did not observe this difference in the dual-stimulus task (Experiment 2). This difference might be due to a difference in task load. Durations appear shorter under high task load situations [38]. Thus, if summing durations of visual and auditory events (i.e., across-modality summation) had higher task demands, this might result in a smaller summation bias in the across-modality conditions.

### Possible processes underlying the overestimation bias

Why does duration summation lead to a larger overestimation bias? Even the estimation of a single temporal duration is not necessarily veridical. For example, brighter, larger, novel, moving, or complex objects appears to last longer [2]. However, in our study the stimuli were identical in Experiment 1 and 2, so these stimulus-driven biases cannot account for the summation bias. Cognitive load also modulates duration estimation [38]. In our study, the summation task may have required more cognitive resources than the dual-stimulus task. However, in the prospective paradigm (where participants know that they will be asked to judge temporal duration), high cognitive load has been shown to lead to duration underestimation, not overestimation [38]. It is noteworthy that the summation task may have required fewer cognitive resources than simply storing two separate durations. If this is the case, the cognitive load account would explain the larger overestimation bias in the summation task. Future studies testing the effect of cognitive load on the summation task are needed to clarify this issue. Another possible factor is attention to the timing task, or arousal more generally. Attended objects seem to last longer than unattended objects [2,3,35,36]. In addition, high arousal pictures are judged as lasting longer than low arousal pictures [39]. These studies examined attention and arousal induced by stimuli, but changes in mental state induced by task requirements may have similar effects. In our case, the mental summation task might make participants attend more to the timing task or lead to higher arousal because the summation task requires constructing a specific duration representation to manipulate rather than simply reproducing input duration. This account is not specific to mental summation; additional studies are necessary to test the arousal/attention modulation account. However, we also propose specific effects of mental summation, such as “operational momentum,” as causing overestimation. We discuss this hypothesis in the following section.

### Toward a unified theory of mental manipulation of magnitudes

The overestimation bias in duration summation may arise from processes that are not specific to time estimation. It is known that adding two numbers leads to an overestimation bias (*operational momentum* [15–18]). Overestimation is associated with spatial representations of numbers and is explained by a process analogous to another spatial phenomenon (*representational momentum* [40]). In addition, some studies have shown overestimation in summing two spatial magnitudes [19,20]. The overestimation of number, space, and duration representations might share common processes that are explained by the operational momentum hypothesis. Furthermore, recent studies have suggested that humans may use common cortical mechanisms to quantitatively represent time, space, and number (A Theory of Magnitude; AToM [21]). If magnitude representations are mediated by a common mechanism, it is plausible that the processes involved in magnitude manipulation are also shared across domains. The supra-modal nature of duration summation supports this notion. Taken together, mental summation of magnitudes may rely on common processes and lead to an overestimation bias irrespective of the stimulus dimension. If this is the case, AToM may be extended from representation to manipulation (A Theory of Magnitude Manipulation; AToMM).

## Supporting Information

S1 Table Means and standard deviations of (normalized) reproduced duration ass a function of experimental condition. (XLSX)

## References

[1] Buhusi CV, Meck WH. What makes us tick? Functional and neural mechanisms of interval timing. Nat Rev Neurosci. 2005;6: 755–765. 10.1038/nrn1764 16163383

[2] Eagleman DM, Pariyadath V. Is subjective duration a signature of coding efficiency? Philos Trans R Soc Lond, B, Biol Sci. The Royal Society; 2009;364: 1841–1851. 10.1098/rstb.2009.0026 19487187 PMC2685825

[3] Grondin S. Timing and time perception: a review of recent behavioral and neuroscience findings and theoretical directions. Atten Percept Psychophys. 2010;72: 561–582. 10.3758/APP.72.3.561 20348562

[4] Ivry RB, Schlerf JE. Dedicated and intrinsic models of time perception. Trends Cogn Sci (Regul Ed). Elsevier; 2008;12: 273–280. 10.1016/j.tics.2008.04.002 PMC4335014 18539519

[5] Droit-Volet S, Wearden J, Delgado-Yonger M. Short-term memory for time in children and adults: A behavioral study and a model. J Exp Child Psychol. 2007;97: 246–264. 10.1016/j.jecp.2007.02.003 17543328

[6] Grondin S. Overloading temporal memory. J Exp Psychol Hum Percept Perform. 2005;31: 869–879. 10.1037/0096-1523.31.5.869 16262484

[7] Nobre AC, O'Reilly J. Time is of the essence. Trends Cogn Sci (Regul Ed). Elsevier; 2004;8: 387–389. 10.1016/j.tics.2004.07.005 15350237

[8] Rao SM, Mayer AR, Harrington DL. The evolution of brain activation during temporal processing. Nat Neurosci. 2001;4: 317–323. 10.1038/85191 11224550

[9] Takahashi K, Watanabe K. Short-term memory for event duration: modality specificity and goal dependency. Atten Percept Psychophys. Springer-Verlag; 2012;74: 1623–1631. 10.3758/s13414-012-0347-3 22810560

[10] Bryce D, Seifried-Dübon T, Bratzke D. How are overlapping time intervals perceived? Evidence for a weighted sum of segments model. Acta Psychol (Amst). 2015;156: 83–95. 10.1016/j.actpsy.2014.12.007 25703484

[11] Fortin C, Massé N. Expecting a break in time estimation: attentional time-sharing without concurrent processing. J Exp Psychol Hum Percept Perform. 2000;26: 1788–1796. 11129374 10.1037//0096-1523.26.6.1788

[12] Fortin C, Bédard M-C, Champagne J. Timing during interruptions in timing. J Exp Psychol Hum Percept Perform. 2005;31: 276–288. 10.1037/0096-1523.31.2.276 15826230

[13] Gaudreault R, Fortin C, Macar F. Contrasting effects of interference and of breaks in interval timing. Acta Psychol (Amst). 2010;133: 3–16. 10.1016/j.actpsy.2009.07.013 19716109

[14] Viau-Quesnel C, Gaudreault R, Ouellet A-A, Fortin C. Making Sense of Timing and Attention: Modality Effect in Timing with a Break. Timing & Time Perception. 2014;2: 129–144. 10.1163/22134468-00002019

[15] McCrink K, Dehaene S, Dehaene-Lambertz G. Moving along the number line: operational momentum in nonsymbolic arithmetic. Percept Psychophys. 2007;69: 1324–1333. 18078224 10.3758/bf03192949

[16] Knops A, Dehaene S, Berteletti I, Zorzi M. Can approximate mental calculation account for operational momentum in addition and subtraction? Q J Exp Psychol (Hove). Routledge; 2014;67: 1541–1556. 10.1080/17470218.2014.890234 24499435

[17] Knops A, Viarouge A, Dehaene S. Dynamic representations underlying symbolic and nonsymbolic calculation: evidence from the operational momentum effect. Atten Percept Psychophys. 2009;71: 803–821. 10.3758/APP.71.4.803 19429960

[18] Pinhas M, Fischer MH. Mental movements without magnitude? A study of spatial biases in symbolic arithmetic. Cognition. 2008;109: 408–415. 10.1016/j.cognition.2008.09.003 18976986

[19] Charras P, Lupiáñez J. Length perception of horizontal and vertical bisected lines. Psychol Res. 2010;74: 196–206. 10.1007/s00426-009-0243-1 19452164

[20] Charras P, Lupiáñez J. The relevance of symmetry in line length perception. Perception. 2009;38: 1428–1438. 19950477 10.1068/p6287

[21] Walsh V. A theory of magnitude: common cortical metrics of time, space and quantity. Trends Cogn Sci (Regul Ed). 2003;7: 483–488. 14585444 10.1016/j.tics.2003.09.002

[22] Chen K-M, Yeh S-L. Asymmetric cross-modal effects in time perception. Acta Psychol (Amst). 2009;130: 225–234. 10.1016/j.actpsy.2008.12.008 19195633

[23] Heron J, Aaen-Stockdale C, Hotchkiss J, Roach NW, McGraw PV, Whitaker D. Duration channels mediate human time perception. Proc Biol Sci. The Royal Society; 2012;279: 690–698. 10.1098/rspb.2011.1131 PMC3248727 21831897

[24] Lapid E, Ulrich R, Rammsayer T. Perceptual learning in auditory temporal discrimination: no evidence for a cross-modal transfer to the visual modality. Psychon Bull Rev. Springer-Verlag; 2009;16: 382–389. 10.3758/PBR.16.2.382 19293111

[25] Noulhiane M, Pouthas V, Samson S. Is time reproduction sensitive to sensory modalities? European Journal of Cognitive Psychology. Routledge; 2009;21: 18–34. 10.1080/09541440701825981

[26] Ortega L, Lopez F, Church RM. Modality and intermittency effects on time estimation. Behav Processes. 2009;81: 270–273. 10.1016/j.beproc.2009.02.009 19429221

[27] Rattat A-C, Picard D. Short-term memory for auditory and visual durations: evidence for selective interference effects. Psychol Res. 2012;76: 32–40. 10.1007/s00426-011-0326-7 21373945

[28] Brainard DH. The Psychophysics Toolbox. Spat Vis. Brill; 1997;10: 433–436. 9176952

[29] Pelli DG. The VideoToolbox software for visual psychophysics: transforming numbers into movies. Spat Vis. 1997;10: 437–442. 9176953

[30] Kleiner M, Brainard D, Pelli D. What's new in Psychtoolbox-3? Pion Ltd; 2007 pp. 0–0.

[31] Goldstone S, Lhamon WT. Studies of auditory-visual differences in human time judgment. 1. Sounds are judged longer than lights. Percept Mot Skills. Ammons Scientific; 1974;39: 63–82. 10.2466/pms.1974.39.1.63 4415924

[32] Bueti D, Buonomano DV. Temporal Perceptual Learning. Timing & Time Perception. 2014;2: 261–289. 10.1163/22134468-00002023

[33] Allman MJ, Teki S, Griffiths TD, Meck WH. Properties of the internal clock: first- and second-order principles of subjective time. Annu Rev Psychol. 2014;65: 743–771. 10.1146/annurev-psych-010213-115117 24050187

[34] Hagura N, Kanai R, Orgs G, Haggard P. Ready steady slow: action preparation slows the subjective passage of time. Proc Biol Sci. The Royal Society; 2012;279: 4399–4406. 10.1098/rspb.2012.1339 PMC3479796 22951740

[35] Enns JT, Brehaut JC, Shore DI. The duration of a brief event in the mind's eye. J Gen Psychol. Taylor & Francis Group; 1999;126: 355–372. 10.1080/00221309909595371 10555865

[36] Seifried T, Ulrich R. Exogenous visual attention prolongs perceived duration. Atten Percept Psychophys. 2011;73: 68–85. 10.3758/s13414-010-0005-6 21258910

[37] Matthews WJ. How does sequence structure affect the judgment of time? Exploring a weighted sum of segments model. Cogn Psychol. 2013;66: 259–282. 10.1016/j.cogpsych.2013.01.001 23395774

[38] Block RA, Hancock PA, Zakay D. How cognitive load affects duration judgments: A meta-analytic review. Acta Psychol (Amst). 2010;134: 330–343. 10.1016/j.actpsy.2010.03.006 20403583

[39] Gil S, Droit-Volet S. Emotional time distortions: the fundamental role of arousal. Cogn Emot. Taylor & Francis Group; 2012;26: 847–862. 10.1080/02699931.2011.625401 22296278

[40] Freyd JJ, Finke RA. Representational momentum. Journal of experimental psychology Learning, memory, and cognition. American Psychological Association; 1984;10: 126–132.

